# Circulating angiogenic progenitor cell apoptosis in Post-COVID-19 syndrome

**DOI:** 10.1016/j.ijcha.2025.101866

**Published:** 2026-01-08

**Authors:** Julia M. Kröpfl, Christoph Hauser, Luca Beugger, Henner Hanssen, Fabian Schwendinger, Arno Schmidt-Trucksäss

**Affiliations:** Division of Sport and Exercise Medicine, Department of Sport, Exercise and Health, University of Basel, Grosse Allee 6, 4052 Basel, Switzerland

**Keywords:** Circulating progenitor cell, Flow-mediated dilation, Retinal vessel analysis, Post-COVID-19 syndrome, Long-COVID

## Abstract

**Background:**

Cellular endothelial dysfunction in patients recovering from Coronavirus disease 2019 (COVID-19) remains poorly understood. This study examined circulating angiogenic progenitor cells (CAC) and mature endothelial cells (CEC) in individuals with persistent symptoms following hospitalization for COVID-19 (PH-PCS) at ≥ 18-months post-infection.

**Methods:**

We compared PH-PCS (n = 14) to matched controls without symptomatic COVID-19 (n = 7). Examinations included macro- and microvascular structure and function and the analysis of CAC and CEC using flow cytometry.

**Results:**

Estimates indicated somewhat lower apoptotic CAC concentrations (mean difference[md] [95 %CI] = 0.050 cells/µl [0.003, 0.137], p = 0.084) and proportions (% total CAC, 7.7 percentage points (pp) [0.3, 12.9], p = 0.066) in patients compared to controls, though estimates were imprecise. Similar results were observed for apoptotic CEC concentrations (1.202 cells/µl [0.040, 7.518], p = 0.066) and proportions (% total CEC, 2.7 pp [0.2, 23.8], p = 0.048). Live CAC (−7.6 pp [-12.7, −1.1], p = 0.084) and live CEC proportions (−4.9 pp [–23.7, −0.3], p = 0.042) were somewhat enhanced in PH-PCS. Brachial-arterial flow-mediated dilation (baFMD) and retinal vessel imaging parameters showed little evidence for differences between groups, except for maximal arteriolar constriction, where estimates suggested on average higher values in PH-PCS (md [95 %CI] = 1.64 [0.050, 3.63], p = 0.084), but estimates were uncertain. Pooling PH-PCS and controls, correlations were observed between reduced baFMD and both elevated total CEC concentrations (ρ = -0.56, p = 0.038) and decreased apoptotic CAC proportions (ρ = 0.56, p = 0.042).

**Conclusions:**

This study suggests the possibility of unbalanced CAC and CEC apoptosis in PH-PCS, but with uncertain magnitude. The findings might inform hypothesis generation for future studies on (cellular) endothelial function in PH-PCS.

## 1 Introduction

Following infection with severe acute respiratory syndrome coronavirus 2 (SARS-CoV-2), millions of individuals worldwide have developed persistent symptoms such as fatigue and reduced exercise capacity [1]. This condition, termed Post-COVID-19 Syndrome (PCS), is characterized by signs and symptoms that develop during or after infection with COVID-19, persist for more than 12 weeks, and cannot be explained by an alternative diagnosis [2]. Increasing evidence suggests that the pathophysiology of PCS is closely linked to the vascular injury that occurs during acute COVID-19.

During acute infection, hyperinflammation, hypercoagulability, and immune dysregulation induce widespread vasculopathy, leading to endothelial dysfunction, and thromboembolic events [3]. Such vascular disturbances may not resolve completely after recovery, contributing to the chronic symptoms seen in PCS. Indeed, convalescent patients show impaired macrovascular function [4], [5], while persistent alterations in microvascular structure and function have been associated with PCS severity, chronic inflammation, and fatigue [6], [7]. These functional impairments reflect a continuum of endothelial damage extending from the acute phase into long-term recovery, suggesting that disrupted vascular homeostasis is a central mechanism linking acute infection to PCS.

At the cellular level, endothelial injury and reduced repair capacity are also evident in PCS [8], [9] and can be traced back to the acute infection; In general, endothelial health relies heavily on the regenerative capacity of circulating angiogenic progenitor cells (CAC), which contribute to vascular repair and maintenance through differentiation and paracrine signalling [10], [11]. SARS-CoV-2 can directly impact these processes: ACE2 expression on endothelial and hematopoietic precursors renders them susceptible to infection [12], while viral-induced changes in the circulating proteome alter pathways involved in coagulation, inflammation, and progenitor cell function [13], [14] disrupting macro- and microvascular endothelial regeneration, with SARS-CoV-2-induced transcriptomic alterations being passed on to mature endothelial cells [15]. Consequently, impaired CAC activity may compromise endothelial regeneration and perpetuate vascular dysfunction in PCS. This dysregulation extends to mature endothelial cells, which exhibit sustained activation and detachment not only during the acute infection, but also the convalescent period − detectable as circulating endothelial cells (CEC) [8]. The persistence of endothelial and immune cell activation, together with epigenetic reprogramming of mononuclear cells [16] may therefore underlie the vascular and systemic sequelae of PCS.

Apoptosis represents another key intersection between viral infection, immune regulation, endothelial health, and long-term consequences. While controlled apoptosis eliminates infected cells and limits viral spread during acute COVID-19, excessive or dysregulated apoptosis can exacerbate endothelial injury and impair vascular regeneration [17]. Heightened apoptotic activity has been identified as a predictor of PCS [17], [18], underscoring the contribution of sustained cellular stress and impaired survival pathways to long-term vascular dysfunction.

Thus, preserving cellular viability and endothelial integrity may be critical for mitigating the chronic manifestations of PCS [19], but integrative studies combining surrogate markers of endothelial dysfunction across cellular, microvascular, and macrovascular levels remain scarce.

Therefore, this study aimed to 1) quantify cellular endothelial dysfunction in individuals with persistent symptoms following hospitalization for COVID-19 (PH-PCS) compared to controls and 2) associate cellular markers of endothelial dysfunction with micro- and macrovascular parameters. It was hypothesized that patients with PH-PCS would exhibit reduced micro- and macrovascular health, greater endothelial damage and less regenerative capacity, increased CEC and decreased CAC levels compared to controls. CEC and CAC apoptosis in PH-PCS would be enhanced.

## 2 Materials and methods

### 2.1 Study design

Patients ≥ 40 years who, at the time of study examination, were ≥ 18 months post–COVID-19 hospitalization with SARS-CoV-2 infection confirmed by PCR- or antigen test, and reported at least one of five commonly described persistent symptoms (post-exertional malaise, fatigue, dyspnoea, exercise intolerance, cognitive impairment), were recruited. Recruitment was conducted at the University Hospital of Basel (Switzerland), including the COVID-19 testing centre, Clinic Barmelweid (Switzerland), and Cantonal Hospital Olten (Switzerland). Recruitment letters were sent to all patients registered in the hospital databases with confirmed SARS-CoV-2 infection within the 16 months preceding the start of recruitment, allowing sufficient time to plan study visits. Patients with multiple past infections were eligible if they fulfilled all inclusion and no exclusion criteria. Controls ≥ 40 years without history of symptomatic COVID-19, vaccinated with ≥ 2 doses, were matched 1:2 by sex, age, and Charlson comorbidity index, which balances overall health burden rather than specific diseases. Exclusion criteria included: language barriers, psychological issues, dementia, pregnancy, breastfeeding, contraindications to exercise testing (e.g., uncontrolled hypertension, epilepsy, active cancer treatment, unstable angina, severe valve disease, acute infection, or musculoskeletal injuries), or recent participation in other clinical trials or prior involvement in this study. The study was conducted at the University of Basel (DSBG), Switzerland, between 03/2023 and 08/2023 in accordance with the Declaration of Helsinki, approved by EKNZ (2021–01039), and registered at clinicaltrials.gov (NCT05118711). Participants fasted ≥ 6h, avoided exercise ≥ 24 h, alcohol/caffeine ≥ 12 h, tobacco ≥ 6h, and emptied their bladder before testing. All participants had a medical history, PCS symptom questionnaire [20], physical exam, and resting ECG.

### 2.2 Cardiorespiratory fitness

Peak oxygen uptake (V̇O_2_peak) was assessed using cardiopulmonary exercise testing on a cycle ergometer (Ergoselect 200, Ergoline, Bitz, Germany). Depending on their fitness level, participants completed one of three ramp protocols. All began with 3 min of rest and a 3-minute warm-up at 20 W for protocols 1 and 2 and 50 W for protocol 3, followed by a continuous workload increase until exhaustion and a 3-minute cool-down at the same intensity as the warm-up [21]. Protocols 1, 2, and 3 used 10, 15, and 20 W/min increases, respectively, until symptom limitation or in controls until maximum voluntary exertion. V̇O_2_peak was determined as the highest 30-second averaged V̇O_2_ value during the test [22]. In the absence of a plateau in V̇O_2_, age-specific cut-points were used to verify achievement of V̇O_2_peak (age group 20 to 39 yrs / RERmax ≥ 1.13; 40 to 59 yrs / ≥ 1.10; 60 to 69 yrs / ≥ 1.06) [21].

### 2.3 Macro- and microvascular assessments

Macrovascular function as brachial-arterial flow-mediated dilation (baFMD) was assessed semi-automatically with an ECG-guided high-resolution B-mode ultrasound system (UNEX EF 38G, UNEX Corp., Nagoya, Japan), following current guidelines [23]. Measurements were taken in a dark, temperature-controlled room after 15 min of supine rest, with patients wearing noise-canceling headphones. A 10-MHz H-shaped probe was used to acquire short- and long-axis images of the right brachial artery, with continuous automatic probe adjustment. The cuff was placed 1–2 cm proximal to the cubital fossa and 5–10 cm distal to the probe. Arterial diameter was recorded for 10 s before a 5-min occlusion at SBP + 50 mmHg, again during the last 60 s of occlusion, and continuously for 3 min postdeflation, with ECG-guided end-diastolic values used for analysis. Final baFMD analysis was done by two well-trained independent investigators and results were averaged.

Evaluation of microvascular structure and function involved the use of static retinal vessel imaging analysis (SVA, Topcon TRC NW8; Visualis 3.0, iMEDOS Health GmbH, Jena, Germany; Vesselmap 2, Visualis, iMEDOS Health GmbH) to measure the central retinal venular equivalent (CRVE), central retinal arteriolar equivalent (CRAE), and the arteriolar-venular ratio (AVR), while dynamic retinal vessel analysis (DVA, ZeissFF450, DVA®; IMEDOS Systems GmbH, Jena, Germany) included the measurement of arteriolar and venular flicker-light induced maximal dilatation response (aFID, vFID), and maximal arteriolar constriction (aCON), as previously described [24]. Retinal vessel analysis was routinely performed on the right eye. Participants with glaucoma, cataract, or other ocular conditions impairing reliable assessment were excluded from static and dynamic retinal vessel analyses.

### 2.4 Cell analyses

**Fig. 1:**
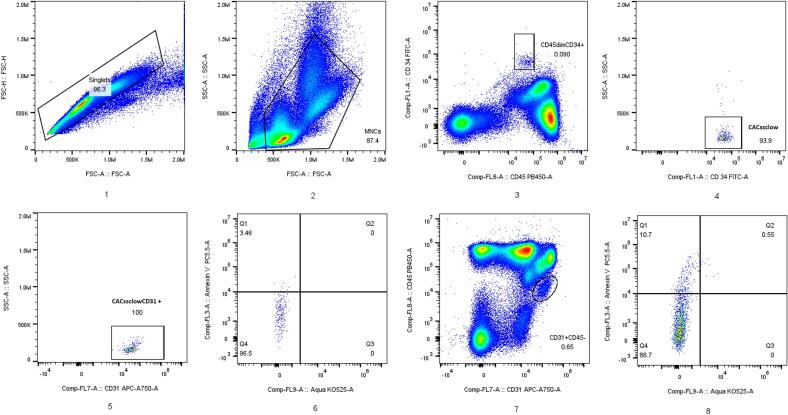
Exemplary flow cytometry gating. After doublet exclusion (1), mononuclear cells (MNC; lymphocytes + monocytes) were gated by forward/side scatter (2), excluding debris. MNC were analyzed for CAC (3–5) or CEC (7), and for live/dead/apoptotic states (6: CD34+/CD45dim/SSClow/CD31+, 8: CD31+/CD45–). Live cells were Q4 (AnnexinV–/Aqua–); apoptotic cells were early (Q1: AnnexinV+/Aqua–) or late (Q2: AnnexinV+/Aqua + ). Results are expressed as % of parent population.

Circulating biomarker analysis was carried out at the in-house molecular-biological laboratory of the DSBG. Blood samples (total volume 7.5 mL, one EDTA tube) were collected by venipuncture of the cubital fossa by the study physician in fasting condition. MNC were immediately isolated by density gradient centrifugation, stained, and analyzed by flow cytometry for relative circulating cell counts using a Cytoflex device (Beckman Coulter, Nyon Switzerland). At least 300,000 MNC were collected. Total, live and apoptotic CAC and CEC were separated using cell surface markers (CD34-PE, clone 4H11, Thermofisher, Schlieren, Switzerland; CD45-FITC, clone HI30, Thermofisher, Schlieren, Switzerland; CD31-APC-Cy7, clone WM59, LucernaChem AG, Lucerne, Switzerland) [10], an apoptosis marker (AnnexinV-PerCP-Cy5.5, BD Biosciences, Allschwil, Switzerland), and a live/dead stain (Aqua, LIVE/DEAD™Fixable Aqua Dead Cell Stain Kit, Thermo Fisher Scientific, Zurich, Switzerland). Relative cell proportions of CAC (CD34+/CD45dim/SSClow/CD31 + ) and CEC (CD31+/CD45-) are given as %MNC and estimates of absolute cell concentrations are described as cells/µl and were calculated by a dual platform-approach [25] after the assessment of whole blood cell counts using a hemolyzer (Sysmex XN-350, Sysmex, Horgen, Switzerland). Live and apoptotic CAC and CEC are expressed as % of the parent population, respectively. Final flow cytometry data were analyzed using FlowJo (LLC, Oregon, USA). Flow cytometry sample analysis is illustrated in Fig. 1. Reticulocytes and related parameters were automatically measured by the same hemolyzer.

### 2.5 Statistics

Data are shown as mean (SD). Based on prior CEC data [26] and a potential drop-out of 10 %, n = 14 participants with PH-PCS and n = 7 with no history of symptomatic COVID-19 were recruited as controls (effect size 1.51, α = 0.05, power = 0.8, allocation ratio 0.5). Parameter distributions were assessed by frequency plots; group comparisons by unpaired t-tests or Mann–Whitney U, reported as mean or median differences (md) with 95 % confidence intervals [95 %CI]. Resulting p-values of related variables (e.g. total CAC and subgroups) were adjusted for multiple testing by the Bonferroni method. Correlations were tested with Pearson or Spearman. Analyses used SPSS v29, GraphPad Prism 8.4.2, and Excel 2016. Statistical significance was assessed at p < 0.05; results were primarily interpreted based on effect sizes, 95 % confidence intervals, and associated uncertainty.

## 3 Results

### 3.1 Study population

**Fig. 2:**
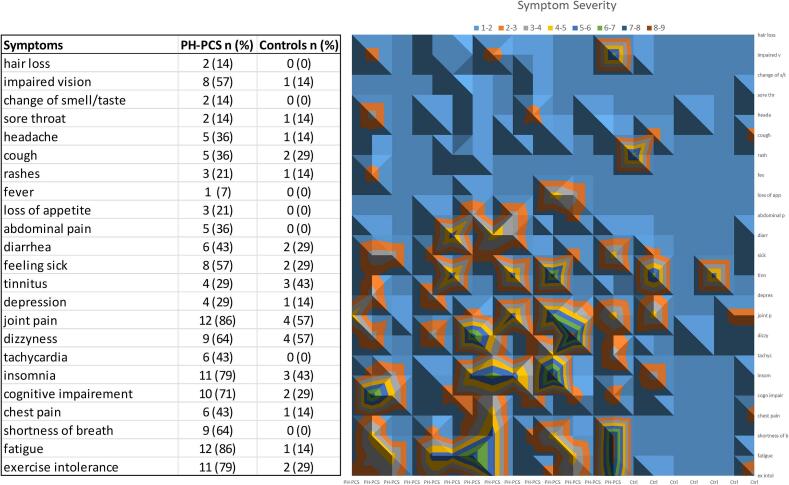
Post-COVID-19 symptoms in the study population. Experienced symptom frequency and contour plot of symptom severity in patients with PH-PCS and matched controls (Ctrl). Colors indicate symptom severity from 1 to 9 with 10 being most severe. Grids without contours mean no symptoms (0).

Patients with PH-PCS (n = 14; 3 females) and matched controls (n = 7; 2 females) were balanced for age (PH-PCS: 62.5(9.9) years, Control: 65.6(8.6) years, md [95 %CI] = 3.2 [-6.1, 12.4], p = 0.482), and Charlson comorbidity index (PH-PCS: 2.3(1.3), Control: 2.4(1.7), md [95 %CI] = 0.1 [-1.3, 1.6], p = 0.843), and showed the following characteristics: blood pressure (PH-PCS: 137(19)/ 88(11) mmHg, Control: 131(11)/ 85(9) mmHg, md_syst_ [95 %CI] = -6 [–23, 10], p_syst_ = 0.424, md_diast_ [95 %CI] = -3 [-13, 7], p_diast_ = 0.517), BMI (PH-PCS: 28.4(5.1) kg/m^2^, Control: 24.4(2.9) kg/m^2^, md [95 %CI] = -4.0 [-8.4, 0.4], p = 0.071). Among patients, two (14.3 %) were ex-smokers, and the rest were non-smokers. Four patients (28.6 %) had higher education, and all reported at least one ongoing symptom (Fig. 2). PH-PCS were not vaccinated before hospitalization (86 %, no disclosure 14 %), had higher average use of antihypertensive, respiratory, cholesterol-lowering, antidiabetic, neurological, and rheumatologic medications and more pre-existing conditions than controls (Supplementary Table 1).

### 3.2 Cardiorespiratory fitness

All participants reached the defined exhaustion criterion, except one patient, whose performance was limited by respiratory and leg discomfort. Comparing the two groups, V̇O_2_peak was lower in patients than controls with effects ranging from small to large (24.6 (6.2) vs. 32.6 (8.6); md [95 %CI] = -8.0 ml/min/kg [-14.8, −1.2], p = 0.024).

### 3.3 Macro- and microvascular health parameters

There was little evidence of group differences in SVA parameters, baFMD, aFID, or vFID (Table 1). Although estimates in PH-PCS were higher or lower, the wide confidence intervals are compatible with positive, null, and negative effects. PH-PCS also showed a somewhat stronger aCON compared with controls (Table 1).

**Table 1:** Macro-, microvascular, and cellular parameters of endothelial dysfunction.

	PH-PCS	Controls	md [95 %CI] (controls – PH-PCS)	p-value*
baFMD (%)	7.5 (4.9)	11.1 (6.0)	3.6 [-2.6, 9.9]	0.454
baDiameter_baseline_ (mm)	4.2 (0.9)	4.4 (0.8)	0.2 [-0.8, 1.2]	1.000
CRAE (µm)	176.7 (10.6)	141.4 (26.5)	−35.29 [-97.23, 26.52]	0.423
CRVE (µm)	218.3 (27.3)	186.6 (40.1)	−31.73 [-75.30, 11.84]	0.411
AVR	0.82 (0.09)	0.76 (0.08)	−0.055 [-0.18, 0.07]	1.000
aFID (%)	3.27 (1.86)	5.47 (0.91)	1.75 [-0.04, 3.53]	0.162
vFID (%)	4.52 (2.50)	6.61 (1.32)	2.09 [-0.93, 5.10]	0.462
aCON (%)	−1.83 (−3.63, −0.85)	−0.19 (−0.85, 0.12)	1.64 [0.050, 3.63]	0.084
Total CEC (cells/µl)	65.607 (42.747)	59.513 (58.824)	−6.093 [-55.288, 43.101]	1.000
Total CEC (% MNC)	2.8 (2.0)	3.1 (2.9)	0.3 [-2.0, 2.6]	1.000
Live CEC (cells/µl)	64.010 (43.554)	52.302 (56.391)	−11.707 [-60.347, 36.933]	1.000
Live CEC (% total CEC)	99.6 (99.4, 99.7)	94.7 (75.9, 97.1)	−4.9 (–23.7, −0.3)	0.042
Total CAC (cells/µl)	1.275 (0.841, 2.120)	0.855 (0.677, 1.134)	−0.420 [-1.229, 0.127]	0.426
Total CAC (% MNC)	0.044 (0.035, 0.088)	0.052 (0.041, 0.065)	0.008 [-0.039, 0.020]	1.000
Live CAC (cells/µl)	1.267 (0.836, 2.047)	0.831 (0.601, 0.986)	−0.437 [-1.244, 0.063]	0.204
Live CAC (% total CAC)	98.9 (98.1, 99.5)	91.4 (86.6, 97.2)	−7.6 (−12.7, −1.1)	0.084

Values are displayed as mean (SD) or median (Q1, Q3). n_patient_ = 14, except for baFMD, CRAE, CRVE, AVR n = 10; aFID, vFID, aCON n = 8; CAC and CEC subgroups n = 12; n_control_ = 7 except for baFMD n = 5; CRAE, CRVE, AVR n = 3; aFID, vFID, aCON n = 4; baFMD, brachial-arterial flow-mediated dilation; CRAE, central retinal arteriolar equivalent; CRVE, central retinal venular equivalent; AVR, arteriolar-venular ratio; aFID, maximum arteriolar flicker light-induced dilation; vFID, maximum venular flicker light-induced dilation; aCON, maximum arteriolar constriction, CEC, circulating endothelial cells; CAC, circulating angiogenic progenitor cells; md [95 %CI], mean or median difference with 95 %-confidence interval; * Bonferroni corrected.

### 3.4 Surrogate parameters of endothelial damage and repair

**Fig. 3:**
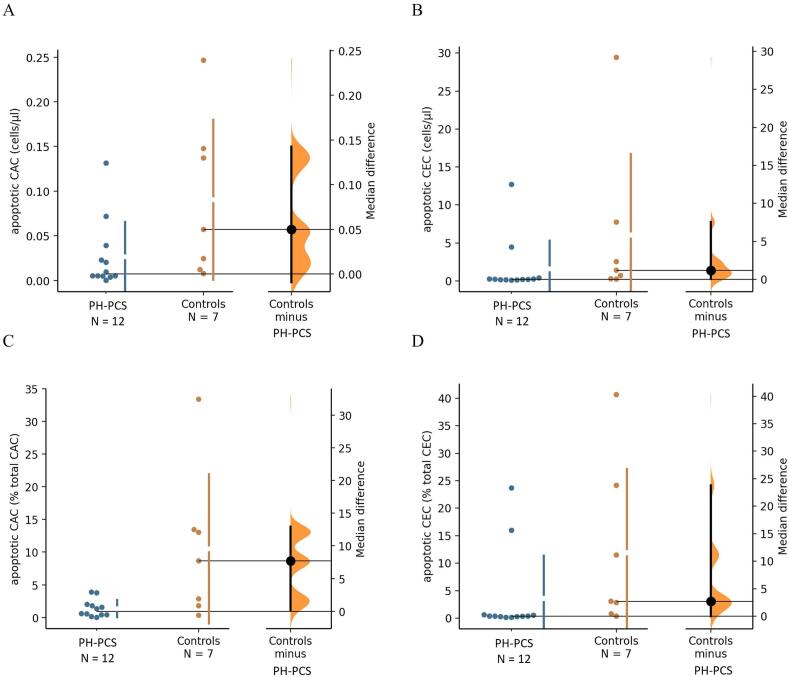
Regulated cell death Apoptosis of CAC and CEC in PH-PCS (n = 12) vs. controls (n = 7) [51]. The unpaired median differences for apoptotic CAC and apoptotic CEC concentration estimates between PH-PCS and Controls are 0.050 cells/µl [95 %CI 0.003, 0.137] and 1.202 cells/µl [95 %CI 0.040, 7.518] with a p-value of 0.084, and p = 0.066, respectively (A, B). Expressed as cell proportions (% parent population), the unpaired median differences for apoptotic CAC and apoptotic CEC between PH-PCS and controls are 7.7 pp [95 %CI 0.3, 12.9], p = 0.066, and 2.7 pp [95 %CI 0. 2, 23.8], p = 0.048, respectively (C, D). Mann-WhitneyU Test was used for group comparison; Color differences indicate patients with Post-COVID-19 (PH-PCS: n = 12) and controls (n = 7).

There were little meaningful differences in total and live CEC as well as CAC between groups when expressed as cell concentrations (Table 1). When expressed as % parent population, estimates of both live CEC (md [95 %CI] = -4.9 percentage points (pp) [–23.7, −0.3], p = 0.042) and live CAC (md [95 %CI] = -7.6 pp [-12.7, −1.1], p = 0.084) were suggestive, but imprecise, of increased values in PH-PCS compared to controls. Also, apoptotic CAC and apoptotic CEC were somewhat reduced in PH-PCS compared to controls when described as cell concentration estimate (md [95 %CI] = 0.050 cells/µl [0.003, 0.137], p = 0.084; md [95 %CI] = 1.202 cells/µl [0.040, 7.518], p = 0.066; both n = 19, Fig. 3, Fig. 3) as well as when described as cell proportion (% total CAC, md [95 %CI] = 7.7 pp [0.3, 12.9], p = 0.066; % total CEC, md [95 %CI] = 2.7 pp [0.2, 23.8], p = 0.048, Fig. 3, Fig. 3).

### 3.5 Blood cell counts

There was suggestive, but imprecise evidence for higher red blood cell counts (RBC) in PH-PCS, while monocytes were increased in PH-PCS relative to controls (Table 2). All other blood cell and reticulocyte parameters showed little evidence for group differences (Table 2).

**Table 2:** Differential blood cell count and reticulocyte parameters.

	PH-PCS	Controls	md [95 %CI] (controls – PH-PCS)	p-value*
WBC, 10^3^/µl	7.48 (1.55)	5.30 (2.23)	−2.18 [-3.96, −0.40]	0.114
RBC, 10^6^/µl	5.24 (0.57)	4.60 (0.51)	−0.64 [-1.19, −0.10]	0.088
Hct, %	45.82 (5.15)	41.46 (5.00)	−4.36 [-9.38, 0.66]	0.340
Hgb, g/l	159.08 (18.99)	144.71 (16.31)	−14.36 [–32.23, 3.50]	0.432
MCV, fl	87.4 (3.1)	90.1 (3.2)	2.67 [-0.42, 5.76]	0.348
Lymphocytes, 10^3^/µl	1.94 (0.56)	1.45 (0.37)	−0.49 [-0.99, 0.01]	0.324
Monocytes, 10^3^/µl	0.66 (0.19)	0.42 (0.04)	−0.23 [-0.35, −0.11]	0.006
Neutrophils, 10^3^/µl	4.65 (1.26)	3.20 (2.07)	−1.46 [-3.01, 0.10]	0.384
Basophils, 10^3^/µl	0.04 (0.02)	0.04 (0.01)	0.00 [-0.01, 0.01]	1.000
Eosinophils, 10^3^/µl	0.19 (0.12)	0.19 (0.13)	0.00 [-0.02, 0.02]	1.000
Neutrophil-to-Lymphocyte ratio	2.19 (1.96, 3.24)	1.70 (1.45, 2.61)	−0.49 [-1.46, 0.44]	0.234
Immature granulocytes, 10^3^/µl	0.049 (0.041)	0.027 (0.011)	−0.022 [-0.056, 0.011]	0.183
Immature granulocytes, %	0.6 (0.4)	0.5 (0.1)	0.1 [-0.2, 0.4]	0.560
Platelets, 10^3^/µl	254.0 (68.9)	234.0 (63.4)	−20.00 [-86.13, 46.13]	0.533
Ret, %	1.50 (0.60)	1.25 (0.47)	−0.25 [-0.80, 0.31]	1.000
Ret, 10^6^/µl	0.079 (0.034)	0.059 (0.027)	−0.020 [-0.052, 0.011]	0.191
IRF, %	9.6 (3.2)	7.8 (3.9)	−1.79 [-5.22, 1.64]	1.000
LFR, %	90.4 (3.2)	92.2 (3.9)	1.79 [-1.64, 5.22]	1.000
MFR, %	8.2 (2.4)	7.0 (3.2)	−1.18 [-3.84, 1.48]	1.000
HFR, %	1.4 (1.2)	0.8 (0.8)	−0.61 [-1.67, 0.44]	1.000
RetHb, pg	32.34 (1.50)	32.34 (1.83)	0.00 [-1.60, 1.60]	0.997

Values are displayed as mean (SD) or median (Q1, Q3). n_patient_ = 13; n_control_ = 7, WBC, white blood cell count; RBC, red blood cell count; Hct, hematocrit; Hgb, hemoglobin; MCV, mean corpuscular volume; Ret, reticulocytes; IRF, immature reticulocyte fraction; LFR, low fluorescence ratio Ret; MFR, medium fluorescent ratio Ret; HFR, high fluorescent ratio Ret; RetHb, reticulocyte hemoglobin; md [95 %CI], mean or median difference with 95 %-confidence interval; * Bonferroni corrected.

### 3.6 Correlations between cell and macro-/ microvascular health parameters

**Fig. 4:**
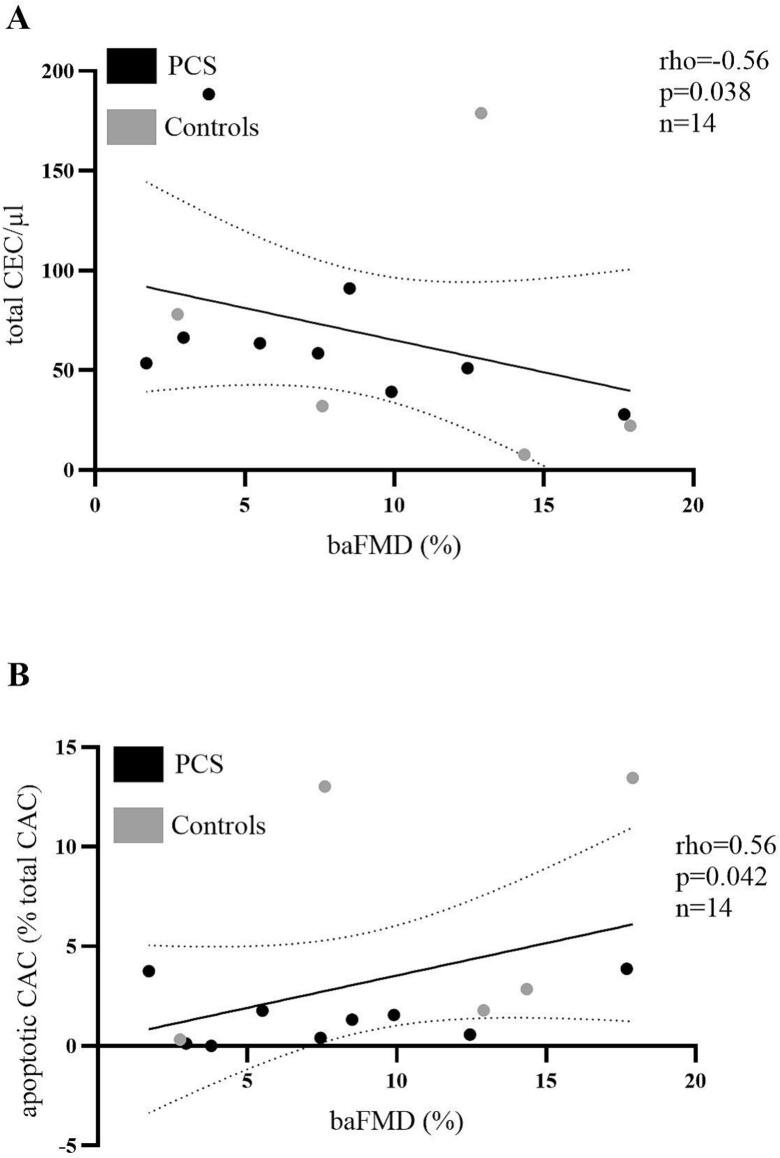
Correlations between macrovascular and cellular parameters of endothelial dysfunction. Association between baFMD and (A) CEC concentration (ρ = -0.56, p = 0.038, n = 14; PH-PCS = 9, controls = 5) and (B) apoptotic CAC (% total CAC, ρ = 0.56, p = 0.042, n = 14) in the pooled dataset. Associations were assessed by Spearman correlation with exact p-values reported.

Pooling all the data, there was a relationship between total CEC concentration estimates and baFMD (ρ = -0.56, p = 0.038, n = 14, Fig. 4A). Lower apoptotic CEC concentration estimates were related to higher CRAE (ρ = -0.645, p = 0.032, n = 11), and better aCON (ρ = 0.673, p = 0.033, n = 10). Total, live and apoptotic CEC proportions did not show any relationship to macro- or microvascular health.

Apoptotic CAC proportions were related to baFMD (ρ = 0.56, p = 0.042, n = 14, Fig. 4B).

Further, CRAE was positively associated with total white blood cells (WBC, r = 0.60, p = 0.039, n = 12), neutrophils (NEU, r = 0.62, p = 0.031, n = 12), and neutrophil-to-lymphocyte ratio (ρ = 0.60, p = 0.031, n = 12), while showing a negative correlation to eosinophils (r = -0.64, p = 0.024, n = 12). For DVA, aFID and vFID were negatively related to NEU (r = -0.68, p = 0.021, n = 11; r = -0.67, p = 0.025, n = 11, respectively). Moreover, aFID was also negatively related to immature granulocytes (IG, r = -0.62, p = 0.043, n = 11), as well as vFID negatively to WBC (r = -0.69, p = 0.019, n = 11).

Total and live CAC concentration estimates were positively associated with IG (r = 0.61, p = 0.005, n = 19; r = 0.60, p = 0.006, n = 19, respectively) and live CAC concentrations with platelets (r = 0.48, p = 0.040, n = 19).

## 4 Discussion

This hypothesis-generating study explores potential differences between patients reporting persistent symptoms ≥ 18 months after SARS-CoV-2–related hospitalization and matched controls. The findings suggest a somewhat lower CAC apoptosis and higher CAC viability in PH-PCS compared with controls, while providing little evidence for differences in total CAC between groups.

During acute COVID-19, regardless of disease severity, endothelial injury peaks [27]. This leads to endothelial shedding and CAC mobilization for vascular repair, increasing viable and apoptotic CAC levels, with apoptosis positively correlating with viral RNA in severe cases [28]. The increase in apoptotic CAC may limit the effectiveness of vascular repair and contribute to disease severity. Conversely, hospitalized patients show reduced hematopoietic progenitors (CD133 + ), a change associated with a 3- to 5-fold higher 1-year mortality risk but not predictive of PCS [29]. This distinction underscores the specific roles of different progenitor cell populations in endothelial repair, namely direct differentiation versus paracrine signalling [10], [11]. Further, apoptotic CEC are significantly reduced in patients with acute COVID-19 compared to healthy controls and negatively related to systemic inflammation [28]. These data indicate that both CAC and CEC regulated cell death is unbalanced during acute infection, even in mild COVID-19. Assessing viability and apoptosis of these cellular markers is hypothesized as a sensitive and specific method for diagnosing COVID-19 severity [30].

While recovered patients might exhibit fewer viable and apoptotic CAC than those acutely infected, levels remain higher than in healthy controls [28]. Apoptotic CEC might be less in recovered patients than controls or those acutely infected [28]. However, convalescent patients discharged after ∼ 15 days show no significant CAC differences from healthy individuals anymore regardless of disease severity, while total CEC remain elevated in those recovering from a moderate disease course indicating a pro-inflammatory and pro-coagulant endothelial state [26].

In contrast to our original hypothesis, our results suggest possibly higher CAC viability and reduced apoptosis compared to controls during PH-PCS, although total CAC showed little evidence for a difference among groups and findings are uncertain. This observation aligns with recent findings of increased CAC colony-forming units being associated with elevated hemoglobin levels and more severe hypoxemia at 3, 6, and 12 months post-infection [31], [32], suggesting enhanced CAC functionality and, by extension, improved viability and reduced apoptotic activity in culture. Our findings build on this evidence by hinting that even ≥ 18 months post-hospitalization, CAC apoptosis might remain unbalanced, though this pattern should be interpreted cautiously. This could reflect epigenetic changes in CAC phenotypes toward more mature subpopulations less prone to apoptosis contributing to a chronic inflammatory environment [33], reduced CAC mobilization pressure and turnover or chronic low grade inflammation decreasing cytotoxic responses that typically drive circulating cell apoptosis during acute infection [34]. A reduction of pro-apoptotic stimuli by intracellular signaling changes and metabolic adaptations, such as the internalization of extracellular vesicles from plasma [35], which are known to influence recipient cell fate and regulate apoptotic pathways through their cargo [36], can also be hypothesized. Although the present findings are associated with substantial uncertainty, previous studies indicate that even modest differences in progenitor cell counts may be clinically relevant. In particular, a one-unit increase in circulating CD34^+^ cells has been associated with a 21 % reduction in all-cause mortality and a 37 % reduction in cardiovascular mortality [37], [38], [39]. It is conceivable that similarly subtle alterations in regulated cell death may also have biological relevance. Taken together, these exploratory observations generate the hypothesis of a potential inverse pattern of CAC apoptosis between the acute phase of infection and a later phase of persistent symptoms, should such a phase develop.

Regarding CEC apoptosis, our findings tentatively indicate somewhat lower apoptotic cell counts and somewhat higher viable CEC proportions in patients ≥ 18 months post-hospitalization, although there was little evidence for a difference of total CEC between PH-PCS and controls. Potential causes of long-term alterations in the endothelial cell death machinery in PCS patients remain speculative at this stage. It may also involve persistent (epi)genetic alterations caused by the original viral infection, hypoxia, or inflammation [9]. It could also be an attempt to improve vascular repair, as a few viable CEC may have properties similar to those of CAC [40].

While a prior study reported lower CRAE, AVR, and vFID in early PCS [7], our findings suggest on average higher CRAE, CRVE, AVR, and aCON, but lower aFID, vFID, and baFMD ≥ 18-months post-hospitalization, though large confidence intervals indicate uncertainty and p-values were not significant.

Interestingly, exploring general systemic influences across the total cohort and pooling data from PH-PCS and controls, we detected reduced CAC apoptosis being associated with worsened macrovascular function. This may reflect that a threshold of CAC apoptosis likely exists – a delicate balance that is necessary for effective endothelial repair without tipping into dysfunction. Excessive CAC apoptosis weakens the response to vascular injury, but too little apoptosis may promote maladaptive repair allowing the survival of dysfunctional or senescent cells [41]. The inverse association between CEC apoptosis and CRAE may mirror a comparable association reflecting a threshold for (dys)function within the microvascular circulation. Our pooled results also suggest a potential association between elevated total CEC and decreased macrovascular function, which may reflect reduced vasodilation capacity being related to increased endothelial shedding; however, all of these findings are exploratory and do not yet establish a causal relationship [19]. Nonetheless, evaluating the survival-to-apoptosis ratio of CAC and CEC may provide clinically valuable insights into the degree of endothelial repair dysfunction and cellular endothelial health, respectively − not only in PCS. This should be looked at in larger randomized clinical trials.

When looking at the mature immune system and pooling all the data, CRAE's positive association with WBC, particularly neutrophils and the neutrophil-to-lymphocyte ratio, and its negative correlation with eosinophils suggest a general relationship between microvascular health and inflammation, which might also be relevant in PCS [42], [43]. However, a *meta*-analysis found retinal venular calibers more affected than arterioles in the general population [44]. Similarly, lower DVA responses in both vessel types in our study correlated with higher NEU or IG levels, reinforcing the higher inflammation-lower microvascular function connection. These results underscore the importance of a holistic approach to endothelial dysfunction in the clinic, considering both cellular and phenotypic biomarkers of vascular health.

This study has several limitations, including the lack of pre-COVID-19 baseline data, strongly varying post-hospitalization sampling times, and the absence of longitudinal follow-up, all of which restrict our ability to draw final conclusions about the long-term vascular impact of post-COVID-19 syndrome. Given the heterogeneity of medication use and the limited sample size, we were also unable to adjust for any potential confounding effects − especially angiotensin-converting enzyme inhibitors/ angiotensin receptor blockers, cholesterol-lowering drugs and antidiabetic medication have been associated with increased CAC numbers or improved cell survival/function [45], [46], [47], [48]. The relatively small sample size further limits statistical power and increases susceptibility to variability. Because of this limited cohort, correlation analyses could only be performed in the pooled sample rather than within the individual groups, which reduces the ability to detect disease-specific associations and may mask group-level differences. Independent confirmation of vascular measures is lacking. In addition, the study relies on intermediate vascular endpoints (baFMD, SVA/DVA parameters, CPC/CEC), which, while widely used, do not directly measure clinical vascular outcomes such as arterial/venous thrombosis [49] or myocardial injury [50] and therefore provide only indirect insights into the underlying pathophysiology. Moreover, several measurements across baFMD, SVA/DVA, and flow cytometry were affected by data loss due to insufficient image quality (baFMD: PH-PCS n = 4, Controls n = 2; SVA/DVA: PH-PCS n = 2, Controls n = 1) or missing data (SVA/DVA: Controls n = 3; flow cytometry: PH-PCS n = 2), further limiting the robustness and completeness of the dataset.

In conclusion, our study did not identify definitive differences in micro- or macrovascular biomarkers between controls and patients with PH-PCS ≥ 18 months after hospitalization for acute SARS-CoV-2 infection. Nevertheless, point estimates suggest potential residual impairments in macrovascular and venular microvascular function, highlighting the need for further investigation. The timing and extent of any persistent vascular alteration may depend on pre-existing factors, such as a patient’s initial vascular age or PCS physiological variability [5]. In addition, these phenotypic changes may be accompanied by modestly increased cellular viability and reduced regulated cell death in circulating endothelial cells and their progenitors. However, these findings are exploratory and hypothesis-generating, and highlight the need for further investigation in larger, well-characterized PCS cohorts to clarify their clinical relevance.

## CRediT authorship contribution statement

**Julia M. Kröpfl:** Conceptualization, Data curation, Formal analysis, Funding acquisition, Investigation, Methodology, Resources, Supervision, Validation, Visualization, Writing – original draft, Writing – review & editing. **Christoph Hauser:** Writing – review & editing, Validation, Methodology, Investigation, Formal analysis, Data curation. **Luca Beugger:** Writing – review & editing, Investigation, Formal analysis. **Henner Hanssen:** Writing – review & editing, Methodology, Investigation, Formal analysis. **Fabian Schwendinger:** Writing – review & editing, Validation, Supervision, Project administration, Methodology, Investigation, Formal analysis, Data curation, Conceptualization. **Arno Schmidt-Trucksäss:** Writing – review & editing, Validation, Project administration, Methodology, Investigation, Data curation, Conceptualization.

## Funding

The study was financially supported by the Freiwillige Akademische Gesellschaft (FAG) Basel [to JMK], and the University of Basel.

## Declaration of competing interest

The authors declare that they have no known competing financial interests or personal relationships that could have appeared to influence the work reported in this paper.

## Data Availability

The datasets generated during the current study are available from the corresponding author on reasonable request.
