# Supplementary material for: Circulating angiogenic progenitor cell apoptosis in Post-COVID-19 syndrome
Source: Int J Cardiol Heart Vasc. 2026 Jan 8;62:101866. doi: 10.1016/j.ijcha.2025.101866 (PMC12813573; doi:10.1016/j.ijcha.2025.101866)
Supplement: Supplementary Data 1 [file mmc1.docx]

**Supplementary Table 1:** Vaccination status, medications, and pre-existing conditions

| **Characteristic** |  |  | **PH-PCS (n=14)** | | **Controls (n=7)** | | |  |
| --- | --- | --- | --- | --- | --- | --- | --- | --- |
| Sex *(No. (%))* | male |  | 11 | (79) | | 5 | (71) |  |
|  | female |  | 3 | (21) | | 2 | (29) |  |
| Age (at inclusion) *(mean (SD) [n])* | |  | 63 | (10) [14] | | 66 | (9) [7] |  |
| Body mass index *(mean (SD) [n])* | |  | 28.4 | (5.1) [14] | | 24.4 | (2.9) [7] |  |
| Charlson comorbidity index | |  | 2.3 | (1.3) [14] | | 2.4 | (1.7) [7] |  |
| Systolic BP (mmHg) *(mean (SD) [n])* | |  | 137 | (19) [14] | | 131 | (11) [7] |  |
| Diastolic BP (mmHg) *(mean (SD) [n])* | |  | 88 | (11) [14] | | 85 | (9) [7] |  |
| Hospital days *(mean (SD) [n])* |  |  | 11 | (11) [14] | | 0 | (0) [7] |  |
| Intensive care *(N (%))* |  |  | 4 | (29) | | 0 | (0) |  |
|  |  |  |  |  | |  |  |  |
| ***Vaccination status (n (%))*** | |  |  |  | |  |  |  |
| ≥ 2 vaccine doses | |  | - | - | | 7 | (100) |  |
| No vaccination prior to hospitalization | |  | 12 | (86) | | - | - |  |
| Additional vaccination after hospital stay | |  | 12 | (86) | | - | - |  |
|  | 0 |  | 2 | (17) | | - | - |  |
|  | 1 |  | 2 | (17) | | - | - |  |
|  | 2 |  | 3 | (25) | | - | - |  |
|  | 3 |  | 1 | (8) | | - | - |  |
|  | 4 |  | 3 | (25) | | - | - |  |
|  | 5 |  | 1 | (8) | | - | - |  |
| No disclosure |  |  | 2 | (14) | | - | - |  |
|  |  |  |  |  | |  |  |  |
| ***Medications (n (%))*** | |  |  |  | |  |  |  |
| ACE/ARB |  |  | 5 | (36) | | 1 | (14) |  |
| Other antihypertensive medication | |  | 1 | (7) | | 0 | (0) |  |
| Respiratory/lung medication |  |  | 4 | (29) | | 1 | (14) |  |
| Beta-blockers |  |  | 0 | (0) | | 1 | (14) |  |
| Antidiabetics |  |  | 7 | (50) | | 0 | (0) |  |
| Diuretics |  |  | 0 | (0) | | 1 | (14) |  |
| Cholesterol-lowering drugs |  |  | 7 | (50) | | 2 | (29) |  |
| Antiplatelet medication |  |  | 2 | (14) | | 1 | (14) |  |
| Neurological medication |  |  | 1 | (7) | | 0 | (0) |  |
| Rheumatologic medication |  |  | 1 | (7) | | 0 | (0) |  |
|  |  |  |  |  | |  |  |  |
| ***Pre-existing conditions (n (%))*** | |  | **10** | **(71)** | | **5** | **(71)** |  |
| Cardiovascular disease |  |  | 4 | (40) | | 1 | (20) |  |
| Diabetes |  |  | 5 | (50) | | 1 | (20) |  |
| Pulmonary ailments |  |  | 3 | (30) | | 1 | (20) |  |
| Neurological issues |  |  | 1 | (10) | | 1 | (20) |  |
| Musculoskeletal impairments |  |  | 2 | (20) | | 1 | (20) |  |
| Cancer |  |  | 0 | (0) | | 1 | (20) |  |
|  | |  |  |  |  | |  |  |
| *PH-PCS* individuals with persistent symptoms following hospitalization for COVID-19, *BP* blood pressure, *ACE* angiotensin-converting enzyme inhibitor, *ARB* angiotensin receptor blocker, *SD* standard deviation, *n* number; | | | | | | | |  |
|  |  |  |  |  | |  |  |  |
